# Dark-field chest x-ray imaging: first experience in patients with alpha1-antitrypsin deficiency

**DOI:** 10.1186/s41747-022-00263-3

**Published:** 2022-03-01

**Authors:** Gregor S. Zimmermann, Alexander A. Fingerle, Bernhard Renger, Karl-Ludwig Laugwitz, Hubert Hautmann, Andreas Sauter, Felix Meurer, Florian Tilman Gassert, Jannis Bodden, Christina Müller-Leisse, Martin Renz, Ernst J. Rummeny, Marcus R. Makowski, Konstantin Willer, Wolfgang Noichl, Fabio De Marco, Manuela Frank, Theresa Urban, Rafael C. Schick, Julia Herzen, Thomas Koehler, Bernhard Haller, Daniela Pfeiffer, Franz Pfeiffer

**Affiliations:** 1 Department of Medicine I, School of Medicine & Klinikum rechts der Isar, Technical University of Munich, Ismaninger Str. 22, 81675 Munich, Germany; 2 Department of Diagnostic and Interventional Radiology, School of Medicine & Klinikum rechts der Isar, Technical University of Munich, Munich, Germany; 3 Chair of Biomedical Physics, Department of Physics & Munich School of BioEngineering, Technical University of Munich, Garching, Germany; 4 Institute of Medical Informatics, Statistics and Epidemiology, School of Medicine & Klinikum rechts der Isar, Technical University of Munich, Munich, Germany; 5 Philips Research, Hamburg, Germany; 6 Institute for Advanced Study, Technical University of Munich, 85748 Garching, Germany

**Keywords:** Alpha1-antitrypsin deficiency, Dark-field x-ray, Pulmonary emphysema, Radiography (thoracic), Tomography (x-ray computed)

## Abstract

**Background:**

Spirometry and conventional chest x-ray have limitations in investigating early emphysema, while computed tomography, the reference imaging method in this context, is not part of routine patient care due to its higher radiation dose. In this work, we investigated a novel low-dose imaging modality, dark-field chest x-ray, for the evaluation of emphysema in patients with alpha1-antitrypsin deficiency.

**Methods:**

By exploiting wave properties of x-rays for contrast formation, dark-field chest x-ray visualises the structural integrity of the alveoli, represented by a high signal over the lungs in the dark-field image. We investigated four patients with alpha1-antitrypsin deficiency with a novel dark-field x-ray prototype and simultaneous conventional chest x-ray. The extent of pulmonary function impairment was assessed by pulmonary function measurement and regional emphysema distribution was compared with CT in one patient.

**Results:**

We show that dark-field chest x-ray visualises the extent of pulmonary emphysema displaying severity and regional differences. Areas with low dark-field signal correlate with emphysematous changes detected by computed tomography using a threshold of -950 Hounsfield units. The airway parameters obtained by whole-body plethysmography and single breath diffusing capacity of the lungs for carbon monoxide demonstrated typical changes of advanced emphysema.

**Conclusions:**

Dark-field chest x-ray directly visualised the severity and regional distribution of pulmonary emphysema compared to conventional chest x-ray in patients with alpha1-antitrypsin deficiency. Due to the ultra-low radiation dose in comparison to computed tomography, dark-field chest x-ray could be beneficial for long-term follow-up in these patients.

## Key points

- Dark-field chest x-ray (CXR) visualises severity of pulmonary emphysema in alpha1-antitrypsin deficiency.
- Dark-field CXR visualises regional distribution of emphysema at low radiation doses.
- Dark-field CXR provides additional information regarding lung ultrastructure.
- Dark-field CXR may complement conventional CXR and chest computed tomography.

## Background

Alpha1-antitrypsin deficiency (AATD) is one of the most common genetic disorders in respiratory diseases. This autosomal recessive disease is associated with an increased risk of pulmonary emphysema and liver disease [1].

Due to the genetically determined dysfunction of antitrypsin, an obstructive lung disease occurs, which is characterised by emphysema with irreversible destruction of the alveolar walls and enlargement of the distal airspaces, leading to a progressive loss of lung function with a limitation of exercise tolerance, quality of life, and lifespan [1]. The classical phenotype in AATD patients is a basal panlobular emphysema [1]. Many affected patients remain undiagnosed, or the definite diagnosis is delayed by many years. In many cases with AATD, the diagnosis is not made before a stage with already progredient emphysema and an irreversible loss of lung function is already present. Stratified screening measures for this disease have not yet been established in most countries.

Conventional chest x-ray (CXR) images are of limited use as a screening tool for emphysema since only indirect signs of emphysema, which are typically not present until later disease stages, can be observed. Early detection, assessment of regional distribution and quantification of emphysema cannot be achieved by conventional chest x-ray imaging [2–4]. Hence, computed tomography (CT) has become the reference standard for imaging of emphysema since many years. On CT scans, emphysema is characterised by abnormally low attenuating lung parenchyma and its local distribution can be assessed visually or by densitometry [5–7].

Different software applications offer a quantification of emphysema based on chest CT scans by using a threshold method [6, 8, 9]. This CT-based quantification can be used in patients with AATD as a longitudinal parameter [10]. However, compared to conventional CXR, a CT scan exposes the patient to a much higher radiation dose (typically 2–7 mSv for a diagnostic chest CT *versus* below 0.05 mSv for CXR on average). Therefore, CT is currently suitable only as a risk-based imaging procedure and not suitable as a general public screening method [11, 12]. Hence, there is a great need for a sensitive imaging technology which allows early detection and long-term follow-up of emphysema without exposing the patients to the high radiation dose delivered by CT.

**Fig. 1:**
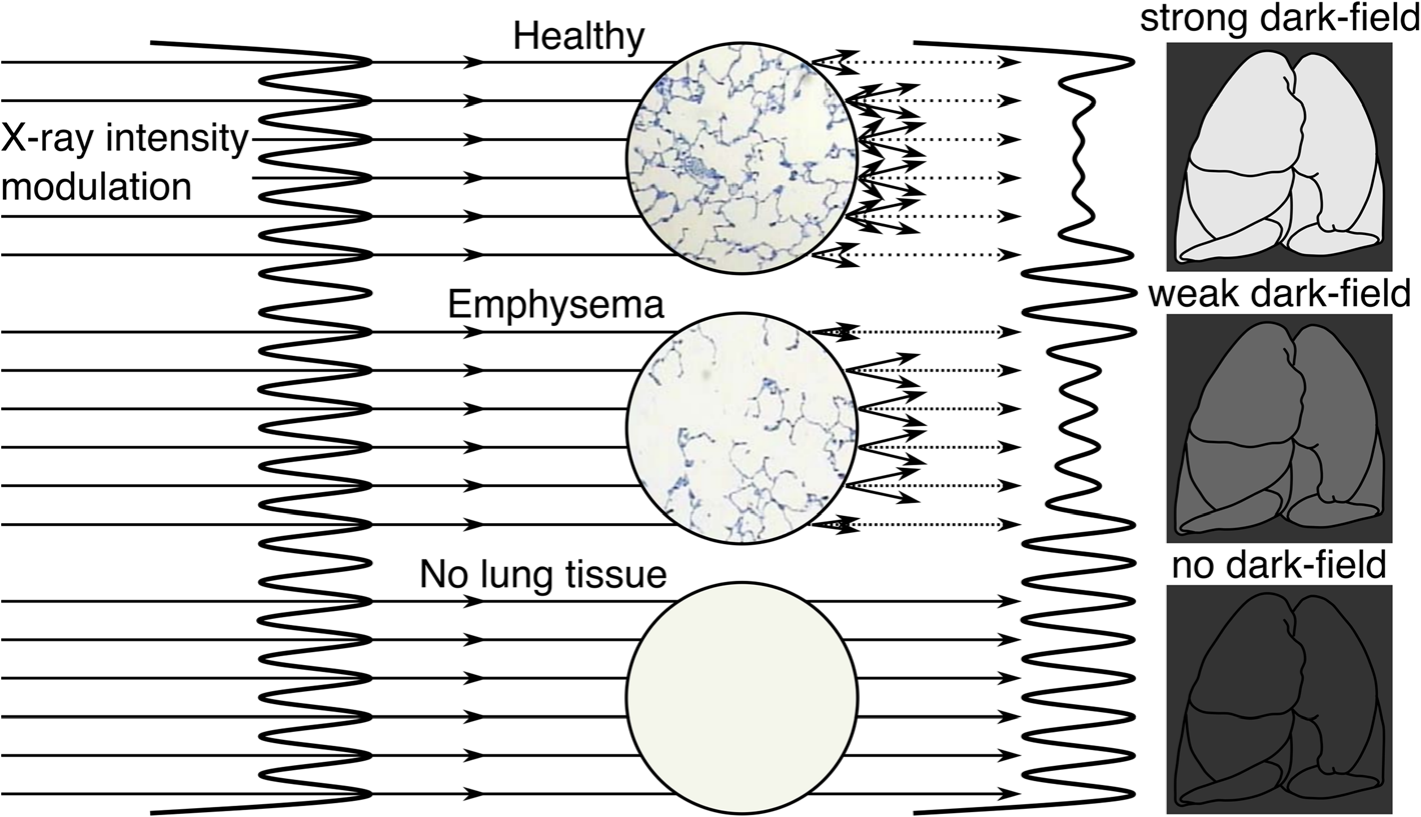
Principle of contrast formation in x-ray dark-field chest radiography. The grating interferometer generates a fine intensity modulation (perpendicular to the beam direction) on the propagating x-ray wave front. Penetrating regions with microscopic interfaces between materials of different refractive indices, this intensity pattern gets distorted as a result of multiple refractions. The dark-field signal intensity is encoded in the reduction of the pattern’s amplitude (fringe contrast). Composed of many inherent interfaces (air *versus* tissue), healthy lung parenchyma induces strong small angle scattering yielding a distinct dark-field signal. On the contrary, emphysematous regions where the respiratory surface is reduced or has already been fully vanished, represent less or no scatter activity resulting in a reduced or zero dark-field signal

Over the last decade, a novel x-ray imaging technology, dark-field radiography, has been developed, which provides information about the microstructural integrity of the lungs [13, 14]. Paving the way for a clinical application, the underlying principle is based on a three-grating interferometer for x-rays, introduced by Pfeiffer et al. [15], which allows the usage of conventional x-ray sources [15]. In conventional x-ray or CT imaging, image contrast is solely based on the attenuation of the x-ray beam when travelling through an object. However, dark-field x-ray imaging exploits wave properties of x-rays for contrast formation by visualising small-angle scattering occurring at interfaces, *e.g.*, air-tissue interfaces in pulmonary alveoli or bone-bone marrow interfaces in trabecular bone. With an increasing number of interfaces, more small-angle scattering occurs, yielding a higher dark-field signal. The principle of contrast formation in x-ray dark-field chest radiography is depicted in Fig. 1.

Intact lung parenchyma is composed of multiple interfaces between air and soft tissue, thus representing a microstructure with varying refractive index. This can induce strong small-angle scattering leading to distinct reduction of the pattern amplitude in dark-field CXR. This results in a bright representation of the lungs in the final image. Emphysematous tissue in contrast provides less respiratory surface as it is characterised by a pathological degradation of alveoli walls. This corresponds to fewer interfaces between air and soft tissue resulting in a reduced dark-field signal in affected regions. Continuing this sequence of decreased numbers of interfaces all the way to a total absence of interfaces, no small-angle scattering can occur in respective objects leading to a zero-signal in the dark-field image [16–20].

In numerous small animal models of pulmonary diseases, dark-field radiography has proven to provide additional, diagnostic relevant information on microstructural changes of the lungs [16–18]. Alveolar destruction, which is characteristic for pulmonary emphysema, leads to a reduction of small-angle scattering. Recent studies demonstrated that emphysema diagnosis, staging, and mapping of local distribution is feasible with dark-field radiography in small animals [4, 14, 21, 22]. Furthermore, the technology has been tested on large animals and cadaveric human bodies [19, 23, 24] and finally been first applied to patients [20].

The aim of our study was to evaluate dark-field chest radiography for the assessment of presence and distribution of emphysema in patients with AATD in comparison to conventional CXR and, if available, CT. Radiation doses for the different imaging modalities were addressed. Whole-body plethysmography was used to measure lung function parameters.

## Methods

### Patient recruitment

In our supraregional outpatient department for patients with AATD, we routinely perform α1-antiproteinase-inhibitor augmentation therapy in non-smoking patients with the genotype PiZZ or PiZ0 and reduced lung function following ATS/ERS statement [1, 25]. For surveillance, we periodically monitor lung volumes by lung function testing. Conventional CXR is performed when clinically indicated. To consider a lung volume reduction, a detailed evaluation by CT is obtained if the patient is an appropriate candidate.

Patient recruitment in the pulmonary outpatient clinic was part of a larger, ongoing, world´s first study at the department of radiology evaluating dark-field CXR for the early detection of emphysema in patients with chronic obstructive pulmonary disease (COPD), including patients with confirmed COPD on spirometry and patients without COPD for reference. To assess whether lung parenchymal changes could be visualised in this rare lung disease, patients with AATD were also included. Data from AATD patients were pooled from the study.

The study was approved by the institutional ethics review board and the Federal Office for Radiation Protection. All participants gave written informed consent.

### Inclusion and exclusion criteria

We included adult patients with confirmed AATD and a patient without AATD with clinically indicated CXR. We excluded patients with any other additional pulmonary disease apart from AATD-associated emphysema, pregnant patients, and patients not capable of giving consent. Due to the size of the grating interferometer prototype, only a limited field of view was studied. Therefore, patients with a body height larger than 182 cm or a body mass index greater than 30 were excluded.

### Study design

Patients included in this preliminary study received dark-field CXR in posteroanterior and lateral projection, in inspiration and expiration. Conventional CXR was acquired in posteroanterior and lateral projection in inspiration. Whole-body plethysmography was performed. One AATD and one healthy patient received CT imaging for clinical reasons to exclude infiltrates or malignancies. Additionally, the CT in the patient with AATD was performed to obtain information regarding distribution of emphysema planning endoscopic lung volume reduction. Results for the four AATD patients were compared to those obtained for the patient without AATD or other pulmonary diseases as a reference (see the “Patient recruitment” section).

### Dark-field radiography

In grating-based dark-field imaging, a high frequency intensity modulation, typically of a few microns in size, is imposed on the x-ray wavefront. If this wave front penetrates an object with high fluctuations of the refractive index on the micron scale, the amplitude of this pattern is reduced because of multiple small-angle scattering. The magnitude of this reduction corresponds to the dark-field signal intensity [22]. Figure 1 schematically illustrates the basic working principle. The mean value of the intensity pattern encodes the conventional attenuation signal which can be reconstructed in addition to the dark-field signal, from the same dataset. Consequently, two perfectly registered images representing different physical interaction principles are obtained in one single acquisition. Detailed information regarding scanning and reading technique were described earlier by our group [26]. A clinical prototype system developed in-house was utilised to acquire dark-field and attenuation thoracic radiography images of the AATD patients and one patient without obstructive lung disease [20]. The system is based on a combination of a three grating x-ray interferometer and standard medical x-ray components such as high voltage generator (Velara, Philips Healthcare, The Netherlands), source (MRC 200 0508 ROT GS, Philips Healthcare, The Netherlands), collimator (MTR 302, Ralco, Milan, Italy), and flat-panel detector (Pixium FE 4343 F, Trixell, Moirans, France). Prior to the scan, the region to be imaged was adjusted according to the patient’s size. Similarly to conventional CXR, the patient was standing upright and was advised to hold breath for the duration of the image acquisition. The acquisition procedure relies on a scanning approach, where an active area of about 42 × 6.5 cm^2^ is scanned across the patient’s thorax in about 7 s.

### Conventional CXR and CT imaging

Attenuation CXRs were obtained using a commercial radiography system (DigitalDiagnost, Philips Medical Systems, Hamburg, Germany) with a tube voltage of 125 kVp. Two patients received a contrast-enhanced chest CT (IQon Spectral CT, Royal Philips, Amsterdam, The Netherlands), as clinically indicated. Tube voltage was 120 kVp and automatic angular tube current modulation was utilised. Images were reconstructed with 0.9-mm slice thickness, high-resolution kernel, and noise suppression level 6 (iDose 6, Royal Philips, Amsterdam, The Netherlands).

### Pulmonary function test (PFT)

All participants underwent a standardised lung function test at our centre according to the European Respiratory Society Recommendations [27, 28]. The PFT consisted of a combination of spirometry with whole-body plethysmography (MasterScreen Body, Jaeger, Wuerzburg, Germany). If applicable, a measurement of diffusing capacity of carbon monoxide (DLCO) was added (MS-PFT, Jaeger, Wuerzburg, Germany).

### Image evaluation

On both dark-field and conventional radiographs, lungs were divided into six regions: right and left lung upper, middle and lower zone (RLUZ, RLMZ, RLLZ, LLUZ, LLMZ, LLLZ, respectively) [24]. On dark-field images, pulmonary signal strength, as a marker for alveolar integrity, was visually graded for each zone separately using a 6-point scale in consensus reading (G.S.Z., A.A.F): 0 (absent), 1 (very low), 2 (low), 3 (moderate), 4 (high), and 5 (very high). Lateral and posteroanterior dark-field images in expiration were included in this evaluation. Conventional radiographs were evaluated regarding the presence of signs of hyperinflation and vascular changes associated with emphysema on a 2-point scale: 0 (absent) and 1 (present). The extent of emphysema in the clinically indicated CT scan of one patient was visualised in a coronal image by thresholding lung density of -950 HU using vendor-specific software (IntelliSpace Portal, Royal Philips, Amsterdam, The Netherlands). The reading was conducted according to a standardised protocol by consensus reading. Due to the small sample size, no multiple readings were conducted.

## Results

### Patient characteristics

We included four patients with a clinical indication for thoracic imaging from our supraregional outpatient department for patients with AATD. The study cohort was part of a larger study evaluating x-ray dark-field radiography in patients with COPD [20]. All patients with AATD of this study collective were included in this work. Patient characteristics are presented in Table 1.

**Table 1:** Characteristics of a healthy patient (#1) and four patients (#2–5) with alpha-1-antitrypsin deficiency (AATD)

Patient characteristics	#1	#2	#3	#4	#5
Gender	Male	Female	Male	Male	Male
Age (years)	33	41	48	73	55
AATD subtype	–	PiZZ	PiZZ	Pi00	PiZZ
Smoking (pack/years, stop)	Active	16, 2017	18, 2014	Never	13, 2009
Medication (year of start)	–	2018	2005	2014	2010
Duration of therapy	–	2	15	6	10
CAT-score	–	26	9	17	19
GOLD stage	–	4D	4A	2B	4B

Medication and duration of therapy refer to alpha-1-antitrypsin augmentation therapy. *CAT* Chronic obstructive pulmonary disease assessment test, *GOLD* Global Initiative for Chronic Obstructive Lung Disease, *PiZZ* Homozygous proteinase Z-alleles, *Pi00* Homozygous 0 alleles

### Pulmonary function test

All patients with AATD had an obstructive lung function. The mean forced expiratory volume in 1 s (FEV1) was 33.1% predicted (the four values being in #2 27.7%, in #3 20.4%, in #4 56.4%, and in #5 27.8%). The mean Tiffeneau-Pinelli index (FEV1/FVC) was 0.42 (the four values being in #2 0.40, in #3 0.35, in #4 0.67, and in #5 0.37). The mean forced vital capacity (FVC) was 63.4% predicted (the four values being in #2 59.7%, in #3 47.1%, in #4 65.2%, and in #5 81.4%). The mean total lung capacity was 129.5% predicted (the four values being in #2 143.8%, in #3 131.3%, in #4 108.2%, and in #5 134.5%). DCLO was obtained in three patients: the mean was 46.7% predicted (the three values being #2 56.1%, in #3 48.2%, and in #5 35.7%).

### Dark-field radiography

**Fig. 2:**
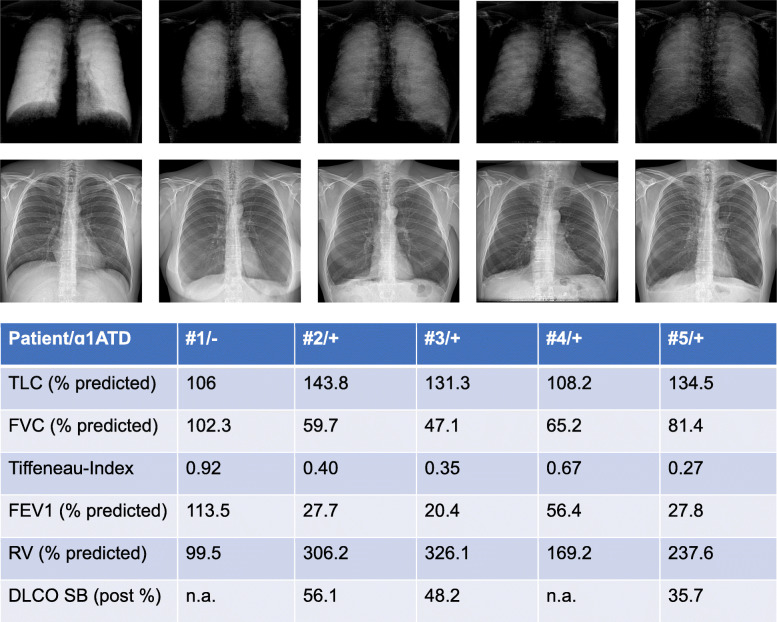
Correlation of dark-field chest x-rays (upper row) and conventional CXR (lower row) of a healthy patient (#1) and four patients (#2–5) with alpha-1-antitrypsin deficiency (AATD) with respiratory parameters obtained by whole-body plethysmography. *TLC*, Total lung capacity; *FVC*, Forced vital capacity; *FEV1*, Forced expiratory volume in 1 s; *RV*, Residual volume; *DLCO SB*, Single breath diffusing capacity of the lungs for carbon monoxide; *% predicted*, compared to reference values; *Post %*, After administration of bronchodilator and compared to reference values

Patient #1 with normal lung condition showed high signal strength (score 4) in upper lung zones and very high (score 5) signal strength in middle and lower lung zones. Signal strength in patient #2 was graded moderate (score 3) in both upper and middle lung zones and low (score 2) in lower lung zones. Patients #3 and #4 demonstrated low signal strength in all lung zones. In patient #5, signal strength was low in lung upper and middle zones and very low in lower zones (shown in Fig. 2).

### Conventional CXR and CT imaging

**Fig. 3:**
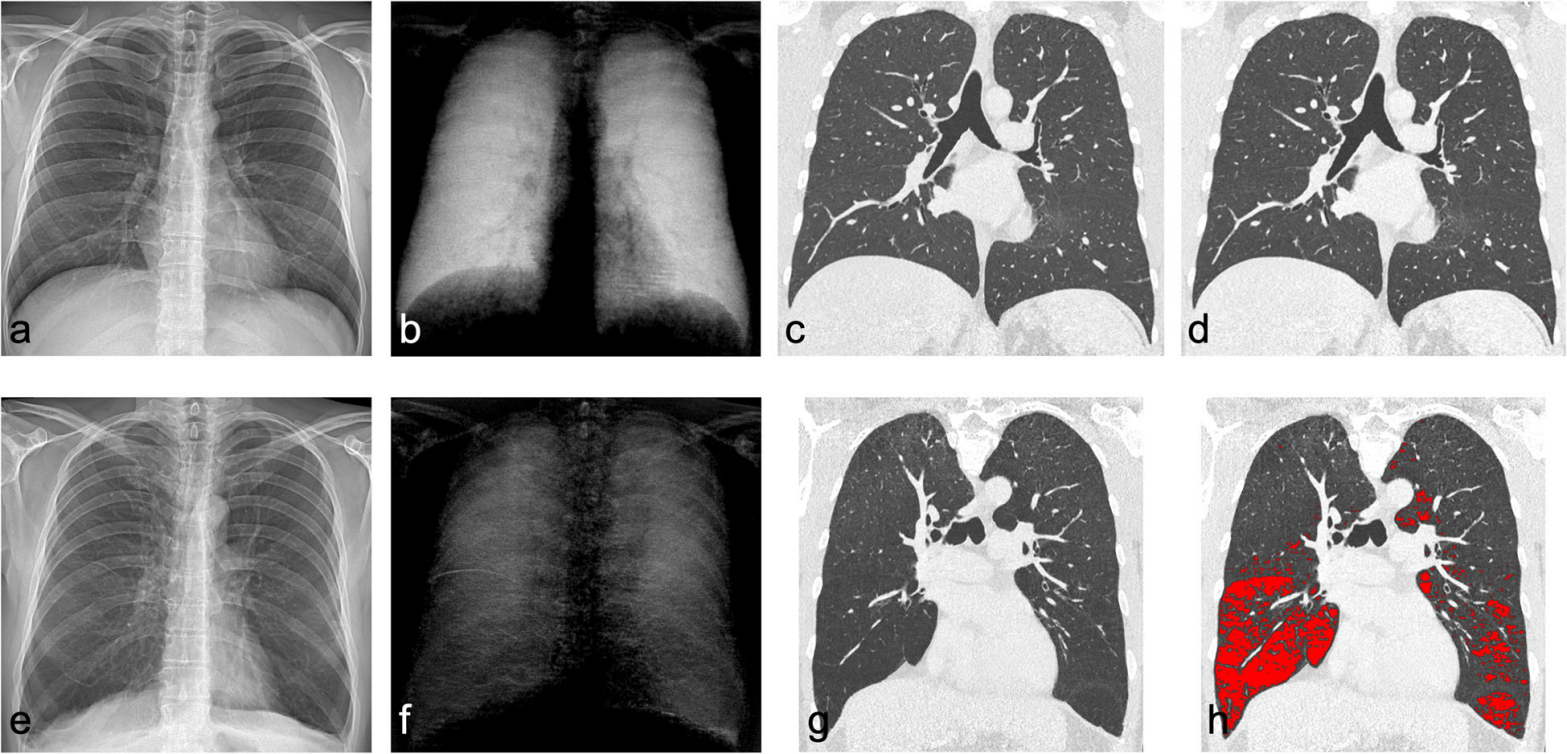
Conventional (**a**) and dark-field chest x-ray (**b**) of a healthy subject and a patient (#5) with alpha1-antitrypsin deficiency (AATD) (**e**, **f**). For both subjects, same window/level settings were applied within conventional and dark-field images, respectively. The dark-field signal is notably reduced in the lungs of the AATD patient (**f**), in particular in lower zones, whereas healthy lungs show a strong homogenous signal over all zones. Corresponding coronal computed tomography (CT) image (**g**) reveals lung parenchymal destruction, highlighted by emphysema overlay (red colour) using a threshold of -950 Hounsfield units (**h**) compared to images of healthy patient (**c**, **d**). Conventional chest x-ray fails to visualise extent of parenchymal disease in the AATD patient (**e**), showing similar transmission in the healthy patient (**a**). Only secondary signs like flattening of diaphragm, increased volume of lungs or widened costophrenic angles point to emphysema (**e**). While the dark-field image exhibits a generally reduced dark-field signal over the entire lung region in contrast to the normal lung, CT only reveals defects in lower regions which correspond to the stronger manifestation of signal reduction in the dark-field radiograph

All patients (#2–5) with AATD showed secondary signs of emphysema in conventional CXRs such as widely spaced ribs and flattened hemidiaphragms. Emphysematous areas defined by low lung density values (lower than -950 HU) showed very low signal strength in the corresponding dark-field image (shown in Fig. 3).

### X-ray radiation dose

Average effective dose for posteroanterior dark-field radiography was 0.049 mSv, 0.032 mSv, 0.047 mSv, and 0.025 mSv for patients #2 to #5.

## Discussion

In this first clinical preliminary study on dark-field CXR in patients with AATD, we showed that dark-field images allow the visualisation of severity and regional distribution of pulmonary emphysema, whereas conventional CXR was not useful for emphysema staging due to its well-known limitations. This pilot study has a demonstrative purpose of the potential application of dark-field imaging. Dark-field images showed a marked signal reduction of the lung parenchyma in AATD patients with advanced emphysema, in comparison to only subtle signs in CXR (Fig. 2). Conversely, conventional CXR found secondary signs of emphysema in our patients. However, these secondary signs reflect only an expression of hyperinflation and no direct signs of emphysema. According to the anatomical definition of emphysema as loss of intact alveoli, dark-field CXR visualised emphysema by reduced dark-field signal strength in these areas in our patients.

In one patient, a very low dark-field signal in the pulmonary lower zones was co-located with emphysema detection in CT that is based on threshold of lung density (Fig. 3). In addition, the dark-field image revealed a general dark-field signal reduction over the entire lung region which was hardly identified by CT densitometry. However, in comparison to CT imaging, which is applied for emphysema quantification and subtyping, dark-field CXR may provide similar information for a fraction of the radiation dose. The radiation dose of dark-field CXR for a reference person is 0.035 mSv which is significantly lower than a CT scan with an average radiation dose of 2–7 mSv [11, 12, 20]. Moreover, in the field of emphysema imaging, dark-field CXR may facilitate early diagnosis and allow screening applications.

Our results are in accordance with previous publications in animals and human body studies. In mice, early stages of emphysema were identified using x-ray dark-field imaging correlating with histological changes, while conventional x-ray could not detect early stages of emphysema [29]. As shown in various studies based on small animal models, the technique has proven to provide additional, diagnostic relevant information on the macro- and micro-structural architecture of lung parenchyma visualising inflammatory, fibrotic, or tumourous changes significantly better than conventional imaging in mice [14, 16–18, 30]. Also, neonatal damage can be visualised in an animal model by x-ray dark-field imaging [31]. Following further preclinical studies on living pigs [19, 23, 32] and cadaveric human bodies [24, 33, 34], the technique has recently been first successfully applied to patients [20]. This clearly demonstrates the feasibility of the technique to comply with clinical boundary constraints, such as field of view, scan time and radiation dose.

A major problem in the early detection of pulmonary emphysema is that emphysematous changes were successfully visualised by CT scans in a large group of smokers even without spirometric abnormality [35, 36]. The degree of emphysema visualised by CT correlates with prognosis of patients with emphysema [35, 36]. However, even low-dose chest CT imaging requires significantly higher radiation exposure than CXR. Thus, an imaging modality is needed that allows early detection of emphysema in patients at risk without the high radiation exposure of CT. In earlier studies, a correlation of DLCO, as a marker for gas exchange surface of the lung, with decreased dark-field signal was described [20]. In our study, we found that despite similar grade of obstruction the dark-field signal strength was higher in patient #2 than in patient #5, assuming an association with the DLCO which was higher in patient #2.

Considering that emphysema due to AATD is usually identified at an advanced stage with progressive damage of the lung, there is an urgent need for additional methods for early detection [1, 37], especially since there is an option of reducing disease progression in these patients by substitution with alpha1 proteinase inhibitor treatment [10, 38]. Since CXR is not appropriate for early detection of structural changes of the lung, dark-field radiography, due to its very low radiation exposure, which is about twice the exposure of a conventional CXR, and its structural information may be particularly well-suited for this purpose.

The main limitations of our study are the single-centre study design and the small sample size. Since there is only one dark-field CXR prototype for use in humans to date, we included patients with AATD at our specialised outpatient clinic to get a first indication whether dark-field radiography has the potential to detect the severity and regional distribution of emphysema in these patients. Further studies are needed to confirm this on larger collectives. In a larger ongoing study in patients with normal lung function and emphysema due to COPD without AATD, dark-field signal strength has shown a higher positive correlation with diffusion capacity, as a marker of intact alveolar surface, than CT-based emphysema quantification [20].

Another limitation is the progressive emphysema of the included patients with AATD in our study which is not representative for early stages of emphysema. The results of patients with early stages of emphysema showing a correlation of dark-field signal strength and DLCO were published recently [20]. Thus, a reduced dark-field CXR signal could indicate that a structural damage already exists despite a normal CXR. This may initiate further diagnostic steps such as pneumological evaluation at an early stage.

The novelty of dark-field radiography as a clinical imaging modality and the lack of experience and training for radiologist readers is a further limitation. However, our radiologists have several years of research experience in reading dark-field images of studies performed in animals and human bodies.

As a conclusion, these preliminary results indicate that dark-field CXR is feasible and can probe the underlying pulmonary microstructure in patients with AATD, which remains inaccessible with currently deployed medical imaging methods at low radiation doses. It may add further information regarding severity and regional distribution of pulmonary emphysema in patients with AATD. The translation of x-ray dark-field imaging to clinical application may have the potential to profoundly change the diagnostics of lung diseases with ultra-low radiation doses compared to CT.

## Data Availability

All data generated or analysed during this study are included in this published article.

## Abbreviations

AATD: Alpha1-antitrypsin deficiency
COPD: Chronic obstructive pulmonary disease
CXR: Chest x-ray
FEV1: Forced expiratory volume in one second
FVC: Forced vital capacity
HU: Hounsfield units
PFT: Pulmonary function test

## References

[1] Miravitlles M, Dirksen A, Ferrarotti I, et al (2017) European Respiratory Society statement: diagnosis and treatment of pulmonary disease in alpha1-antitrypsin deficiency. Eur Respir J 50:1700610. 10.1183/13993003.00610-2017 10.1183/13993003.00610-2017 29191952

[2] Muller NL, Coxson H (2002). Chronic obstructive pulmonary disease. 4: imaging the lungs in patients with chronic obstructive pulmonary disease. Thorax.

[3] Washko GR (2010). Diagnostic imaging in COPD. Semin Respir Crit Care Med.

[4] Meinel FG, Schwab F, Schleede S, et al (2013) Diagnosing and mapping pulmonary emphysema on X-ray projection images: incremental value of grating-based X-ray dark-field imaging. PLoS One 8:e59526. 10.1371/journal.pone.0059526 10.1371/journal.pone.0059526 PMC3608711 23555692

[5] Cavigli E, Camiciottoli G, Diciotti S, et al (2009) Whole-lung densitometry versus visual assessment of emphysema. Eur Radiol 19:1686–1692. 10.1007/s00330-009-1320-y 10.1007/s00330-009-1320-y 19224221

[6] Stoel BC, Stolk J, Bakker ME, Parr DG (2019). Regional lung densities in alpha-1 antitrypsin deficiency compared to predicted values. Respir Res.

[7] Konietzke P, Jobst B, Wagner WL, et al (2018) Similarities in the computed tomography appearance in alpha1-antitrypsin deficiency and smoking-related chronic obstructive pulmonary disease in a smoking collective. Respiration 96:231–239. 10.1159/000489177 10.1159/000489177 29940576

[8] Stoel BC, Stolk J (2004). Optimization and standardization of lung densitometry in the assessment of pulmonary emphysema. Invest Radiol.

[9] Parr DG, Dirksen A, Piitulainen E, Deng C, Wencker M, Stockley RA (2009). Exploring the optimum approach to the use of CT densitometry in a randomised placebo-controlled study of augmentation therapy in alpha 1-antitrypsin deficiency. Respir Res.

[10] McElvaney NG, Burdon J, Holmes M, et al (2017) Long-term efficacy and safety of alpha1 proteinase inhibitor treatment for emphysema caused by severe alpha1 antitrypsin deficiency: an open-label extension trial (RAPID-OLE). Lancet Respir Med 5:51–60. 10.1016/S2213-2600(16)30430-1 10.1016/S2213-2600(16)30430-1 27916480

[11] Mettler FA, Huda W, Yoshizumi TT, Mahesh M (2008). Effective doses in radiology and diagnostic nuclear medicine: a catalog. Radiology.

[12] Larke FJ, Kruger RL, Cagnon CH, et al (2011) Estimated radiation dose associated with low-dose chest CT of average-size participants in the National Lung Screening Trial. AJR Am J Roentgenol 197:1165–1169. 10.2214/AJR.11.6533 10.2214/AJR.11.6533 22021510

[13] Bech M, Tapfer A, Velroyen A, et al (2013) In-vivo dark-field and phase-contrast x-ray imaging. Sci Rep 3:3209. 10.1038/srep03209 10.1038/srep03209 PMC3826096 24220606

[14] Yaroshenko A, Meinel FG, Bech M, et al (2013) Pulmonary emphysema diagnosis with a preclinical small-animal X-ray dark-field scatter-contrast scanner. Radiology 269:427–433. 10.1148/radiol.13122413 10.1148/radiol.13122413 23696682

[15] Pfeiffer F, Bech M, Bunk O, et al (2008) Hard-X-ray dark-field imaging using a grating interferometer. Nat Mater 7:134–137. 10.1038/nmat2096 10.1038/nmat2096 18204454

[16] Yaroshenko A, Hellbach K, Yildirim AO, et al (2015) Improved in vivo assessment of pulmonary fibrosis in mice using x-ray dark-field radiography. Sci Rep 5:17492. 10.1038/srep17492 10.1038/srep17492 PMC4664921 26619958

[17] Hellbach K, Yaroshenko A, Willer K, et al (2016) Facilitated diagnosis of pneumothoraces in newborn mice using x-ray dark-field radiography. Invest Radiol 51:597–601. 10.1097/RLI.0000000000000285 10.1097/RLI.0000000000000285 27603110

[18] Hellbach K, Meinel FG, Conlon TM, et al (2018) X-ray dark-field imaging to depict acute lung inflammation in mice. Sci Rep 8:2096. 10.1038/s41598-018-20193-8 10.1038/s41598-018-20193-8 PMC5794739 29391514

[19] Hellbach K, Baehr A, De Marco F (2018). Depiction of pneumothoraces in a large animal model using x-ray dark-field radiography. Sci Rep.

[20] Willer K, Fingerle AA, Noichl W, et al (2021) X-ray dark-field chest imaging for detection and quantification of emphysema in patients with chronic obstructive pulmonary disease: a diagnostic accuracy study. Lancet Digit Health 3:e733–e744. 10.1016/S2589-7500(21)00146-1 10.1016/S2589-7500(21)00146-1 PMC8565798 34711378

[21] Schleede S, Meinel FG, Bech M, et al (2012) Emphysema diagnosis using X-ray dark-field imaging at a laser-driven compact synchrotron light source. Proc Natl Acad Sci U S A 109:17880–17885. 10.1073/pnas.1206684109 10.1073/pnas.1206684109 PMC3497797 23074250

[22] Meinel FG, Yaroshenko A, Hellbach K, et al (2014) Improved diagnosis of pulmonary emphysema using in vivo dark-field radiography. Invest Radiol 49:653–658. 10.1097/RLI.0000000000000067 10.1097/RLI.0000000000000067 24853070

[23] Gromann LB, De Marco F, Willer K (2017). In-vivo x-ray dark-field chest radiography of a pig. Sci Rep.

[24] Fingerle AA, De Marco F, Andrejewski J (2019). Imaging features in post-mortem x-ray dark-field chest radiographs and correlation with conventional x-ray and CT. Eur Radiol Exp.

[25] American Thoracic Society, European Respiratory Society (2003) American Thoracic Society/European Respiratory Society statement: standards for the diagnosis and management of individuals with alpha-1 antitrypsin deficiency. Am J Respir Crit Care Med 168:818–900. 10.1164/rccm.168.7.818 10.1164/rccm.168.7.818 14522813

[26] Gassert FT, Urban T, Frank M (2021). X-ray dark-field chest imaging: qualitative and quantitative results in healthy humans. Radiology.

[27] Coates AL, Peslin R, Rodenstein D, Stocks J (1997). Measurement of lung volumes by plethysmography. Eur Respir J.

[28] Wanger J, Clausen JL, Coates A, et al (2005) Standardisation of the measurement of lung volumes. Eur Respir J 26:511–522. 10.1183/09031936.05.00035005 10.1183/09031936.05.00035005 16135736

[29] Hellbach K, Yaroshenko A, Meinel FG, et al (2015) In vivo dark-field radiography for early diagnosis and staging of pulmonary emphysema. Invest Radiol 50:430–435. 10.1097/RLI.0000000000000147 10.1097/RLI.0000000000000147 25761095

[30] Scherer K, Yaroshenko A, Bolukbas DA (2017). X-ray dark-field radiography - in-vivo diagnosis of lung cancer in mice. Sci Rep.

[31] Yaroshenko A, Pritzke T, Koschlig M, et al (2016) Visualization of neonatal lung injury associated with mechanical ventilation using x-ray dark-field radiography. Sci Rep 6:24269. 10.1038/srep24269 10.1038/srep24269 PMC4829826 27072871

[32] De Marco F, Willer K, Gromann LB (2019). Contrast-to-noise ratios and thickness-normalized, ventilation-dependent signal levels in dark-field and conventional in vivo thorax radiographs of two pigs. PLoS One.

[33] Willer K, Fingerle AA, Gromann LB, et al (2018) X-ray dark-field imaging of the human lung-a feasibility study on a deceased body. PLoS One 13:e0204565. 10.1371/journal.pone.0204565 10.1371/journal.pone.0204565 PMC6160109 30261038

[34] Sauter AP, Andrejewski J, De Marco F (2019). Optimization of tube voltage in X-ray dark-field chest radiography. Sci Rep.

[35] Lynch DA, Moore CM, Wilson C, et al (2018) CT-based visual classification of emphysema: association with mortality in the COPDGene study. Radiology 288:859–866. 10.1148/radiol.2018172294 10.1148/radiol.2018172294 PMC6122195 29762095

[36] Regan EA, Lynch DA, Curran-Everett D, et al (2015) Clinical and radiologic disease in smokers with normal spirometry. JAMA Intern Med 175:1539–1549. 10.1001/jamainternmed.2015.2735 10.1001/jamainternmed.2015.2735 PMC4564354 26098755

[37] McElvaney NG (2015). Diagnosing alpha1-antitrypsin deficiency: how to improve the current algorithm. Eur Respir Rev.

[38] Ma S, Lin YY, Cantor JO (2016). The effect of alpha-1 proteinase inhibitor on biomarkers of elastin degradation in alpha-1 antitrypsin deficiency: an analysis of the RAPID/RAPID extension trials. Chronic Obstr Pulm Dis.

